# Multimodal integration of magnetic resonance imaging and intracranial electroencephalographic abnormalities in temporal lobe epilepsy surgery

**DOI:** 10.1111/epi.70042

**Published:** 2025-12-22

**Authors:** Csaba Kozma, Jonathan Horsley, Gerard Hall, Callum Simpson, Jane de Tisi, Anna Miserocchi, Beate Diehl, Andrew W. McEvoy, Sjoerd B. Vos, Gavin P. Winston, Yujiang Wang, John S. Duncan, Peter N. Taylor

**Affiliations:** ^1^ CNNP Lab, School of Computing Newcastle University Newcastle Upon Tyne UK; ^2^ Department of Epilepsy, UCL Queen Square Institute of Neurology University College London London UK; ^3^ Western Australia National Imaging Facility University of Western Australia Nedlands Western Australia Australia; ^4^ Centre for Medical Image Computing, Computer Science Department University College London London UK; ^5^ Department of Medicine Queen's University Kingston Ontario Canada; ^6^ Translational and Clinical Research Institute, Faculty of Medical Sciences Newcastle University Newcastle Upon Tyne UK

**Keywords:** epilepsy surgery, gray matter, iEEG, multimodal, superficial white matter

## Abstract

**Objective:**

Precise localization of epileptogenic tissue is critical for successful surgery in drug‐resistant temporal lobe epilepsy (TLE) but is challenging in those requiring intracranial electroencephalography (iEEG). A range of modalities are used for localization, including magnetic resonance imaging (MRI) and EEG, which are typically integrated qualitatively by the clinical team.

**Methods:**

This study quantitatively performed retrospective analysis of three modalities in 40 individuals with TLE who underwent subsequent resective surgery: preoperative diffusion‐weighted MRI, T1‐weighted MRI, and iEEG. Brain abnormalities in gray matter (GM) volume, superficial white matter (SWM) mean diffusivity, and interictal iEEG band power were derived by comparison to 97 MRI controls and 247 subjects with iEEG. We hypothesized that combined abnormalities in GM and SWM could differentiate postsurgical outcomes and adding iEEG abnormalities would improve outcome differentiation.

**Results:**

MRI (union of GM and SWM) abnormalities were primarily concentrated in the ipsilateral hippocampus and inferior temporal regions. Resection of these abnormal regions effectively differentiated seizure‐free outcomes (area under the curve [AUC] = .76, area under the precision–recall curve [AUPRC] = .78, *p* < .01), corroborating previous results from larger TLE cohorts. Adding iEEG abnormalities improved outcome differentiation (AUC = .92, AUPRC = .89, *p* < .01; *z* = 2.01, *p* < .05). MRI abnormalities were more likely to colocalize with iEEG implantation sites (*z* = 6.26, *p* < .01) and iEEG abnormalities (*z* = 4.34, *p* < .01) in individuals with favorable outcomes (International League Against Epilepsy [ILAE] class 1 and 2), but not in those with less favorable outcomes (ILAE class 3+).

**Significance:**

Combining quantitative MRI‐derived GM and SWM abnormalities with interictal iEEG data improves localization of epileptogenic tissue and postsurgical outcome differentiation. Multimodal approaches may offer added value for surgical planning in complex situations.

**Key points**

- Resection of gray and superficial white matter abnormalities together reliably differentiated surgical outcomes, even in complex cases.
- Combining MRI and interictal iEEG abnormalities significantly improved outcome differentiation.
- In cases free from disabling seizures, regions with abnormal gray and superficial white matter were likely to be implanted and resected.

## 1 INTRODUCTION

Approximately half of individuals who undergo epilepsy surgery continue to experience seizures postoperatively, often due to incomplete resection of the epileptogenic zone (EZ).^1^, ^2^, ^3^ Precise localization of the EZ is essential to improve surgical outcomes. This typically requires a multimodal approach, combining seizure semiology, magnetic resonance imaging (MRI), and functional data (e.g., electroencephalography [EEG]).

Gray matter (GM) atrophy is common in TLE and offers important information for localizing the EZ.^4^, ^5^, ^6^ T1‐weighted MRI can detect GM atrophy, which typically involves bilateral temporal and frontal cortices, as well as key subcortical structures including the hippocampus and thalamus.^4^, ^7^, ^8^ The patterns of atrophy reflect the network disruption in focal epilepsy and correlate with disease severity and the likelihood of postoperative seizure freedom.^5^, ^9^

Diffusion‐weighted MRI detects the microstructural diffusion of water molecules across the brain and may infer damage to white matter (WM).^10^ WM abnormalities, particularly in the superficial layer (superficial WM [SWM]), are often lateralized to the seizure onset zone (SOZ)^5^, ^7^ in TLE and may reflect disruptions in local connectivity that contribute to seizure generation.^11^, ^12^ Resection of these abnormalities has been associated with better surgical outcomes.^13^, ^14^, ^15^

Interictal intracranial EEG (iEEG), when compared to patient‐specific normative maps derived from nonepileptogenic regions, can identify abnormal spectral patterns across frequency bands.^16^, ^17^, ^18^ This band power approach can detect pathological regions.^19^, ^20^, ^21^, ^22^

GM, SWM, and interictal iEEG abnormalities provide unique insights, and their combined quantitative analysis remains underutilized. Combining GM and SWM improves outcome differentiation,^8^, ^23^ and integrating all three may improve delineation of epileptic abnormalities.

We tested three hypotheses: (1) MRI (union of GM and SWM) abnormalities can distinguish surgical outcomes even in complex cases requiring iEEG, (2) integrating interictal iEEG band power abnormalities enhances this distinguishability, and (3) resected regions with MRI abnormalities that align with iEEG abnormalities are associated with better outcomes.

## 2 MATERIALS AND METHODS

### 2.1 Subjects

We conducted a retrospective study of 40 individuals with temporal lobe epilepsy (TLE) who underwent presurgical iEEG and temporal lobe resection between 2009 and 2021 at the National Hospital for Neurology and Neurosurgery, London. The study used previously acquired, anonymized data and was approved by the Health Research Authority (University College London Hospitals [UCLH] epilepsy surgery database: 22/SC/0016) and the Database Local Data Monitoring Committee. Individuals who declined the use of their anonymized data for research were excluded. This cohort was a subset of the IDEAS dataset.^24^ Of the 40 patients included in this study, 34 patients overlapped with the cohort described in Kozma et al.^23^ Across the whole cohort, 12 received combined subdural grid and depth electrode implantation, and 28 received depth electrodes only. Electrode implantation strategy was determined clinically based on seizure semiology, imaging findings, and multidisciplinary discussions. All patients had a subsequent temporal lobe resection.

#### 2.1.1 MRI data

Patients were matched with 97 healthy controls who provided written consent (see Table 1). MRI data were acquired using one of two protocols and included anatomical T1‐weighted and diffusion‐weighted imaging (DWI).

**TABLE 1:** Control and patient data by outcome at 1 year.

Characteristic	T1w/dMRI control	iEEG control	$\mathrm{ILA}E_{1,2}$	$\mathrm{ILA}E_{3+}$	Test statistic
*n*	97	247	19	21	
Age of onset, years, median (IQR)	–	–	11 (12.25)	15 (11)	W=268.5,p=.06
Age at scan, years, median (IQR)	39 (20.8)	35 (11.8)	38.3 (14.9)	37.8 (11.2)	K=1.509,p=.60
Sex, male:female	37:60	123:124	10:11	10:9	$\chi^{2}=1.35,p=.61$
Side, left:right	–	–	13:6	6:15	$\chi^{2}=4.85,p=.03$
HS, yes:no	–	–	9:10	12:9	$\chi^{2}=.09,p=.76$

Abbreviations: dMRI, diffusion‐weighted magnetic resonance imaging; HS, hippocampal sclerosis; iEEG, intracranial electroencephalographic; ILAE, International League Against Epilepsy; IQR, interquartile range; T1w, T1‐weighted.

#### 2.1.2 Intracranial EEG

All patients underwent presurgical iEEG to identify SOZs. For this analysis, following the protocol defined in Woodhouse et al.,^25^ patient data were compared to normative iEEG maps derived from 247 participants in the Restoring Active Memory (RAM) dataset.^26^ Whereas these participants were epilepsy patients undergoing iEEG monitoring, data from neurologically healthy individuals are not available for invasive recordings. Therefore, in line with established approaches,^17^, ^18^, ^21^ normative maps were constructed using iEEG signals recorded from electrodes located outside the clinically defined SOZ, representing noninvolved brain regions.

This strategy parallels normative modeling approaches in structural and functional neuroimaging, where individual patient data are compared against normative reference distributions to detect abnormal patterns. The RAM dataset comprises recordings obtained from multiple US epilepsy centers under standardized protocols. For the present study, we used task‐free (resting) intervals to characterize baseline spectral power across electrodes.^26^ There was no overlap between the 40 study patients and the 247 individuals in the iEEG normative map.

#### 2.1.3 Clinical characteristics

Age at epilepsy onset ranged from 1 to 52 years (median = 13 years, interquartile range = 11.62). Hippocampal sclerosis (HS) was present in 52.5% of patients. At 12 months postsurgery, 47.5% achieved International League Against Epilepsy (ILAE) class 1 or 2 seizure outcomes (see Table 1).

### 2.2 Data acquisition

#### 2.2.1 T1‐weighted and diffusion‐weighted MRI

Two acquisition protocols were used. The first cohort (21 patients, 29 controls) was scanned between 2009 and 2013 using a 3‐T GE Signa HDx scanner with standard gradients (40 mT/m^−1^, 150 T/m^−1^·s^−1^) and an eight‐channel coil. T1‐weighted images were acquired with a three‐dimensional (3D) fast spoiled gradient‐echo (FSPGR) sequence (echo time [TE] = 3.04 ms, repetition time [TR] = 37.68 s), producing 170 contiguous 1.1‐mm coronal slices (256 × 256 matrix, .9375 × .9375‐mm in‐plane resolution). DWI was performed using a cardiac‐triggered single‐shot spin‐echo echo planar imaging (EPI) sequence (TE = 73 ms) with 60 axial slices (2.4‐mm thickness, 96 × 96 matrix zero‐filled to 128 × 128, 1.875 × 1.875‐mm in‐plane resolution), 52 diffusion directions (*b* = 1200 s/mm^2^ δ = 21 ms, Δ = 29 ms), and six B0 scans.

The second cohort (19 patients, 68 controls) was scanned between 2014 and 2019 on a 3‐T GE MR750 scanner with higher gradient strength (50 mT/m^−1^, 200 T/m^−1^·s^−1^) and a 32‐channel coil. T1‐weighted images were obtained using a 3D inversion‐recovery FSPGR sequence (TE = 3.1 ms, TR = 7.4 ms, inversion time = 400 ms), yielding 170 contiguous 1‐mm coronal slices (256 × 256 matrix, 1 × 1‐mm in‐plane resolution). DWI used a single‐shot EPI sequence (TE = 74.1 ms, TR = 7600 ms) with 70 axial slices and 115 volumes across four *b*‐values (0, 300, 700, and 2500 s/mm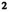, δ = 21.5 ms, Δ = 35.9 ms). Field of view was 256 × 256 mm with a 128 × 128 acquisition matrix, and final voxel size of 2 × 2 × 2 mm.

#### 2.2.2 Intracranial EEG

Electrode contacts were localized to the nearest brain regions using Euclidean distance. Contacts >5 mm from any region, or within WM and >2 mm from GM, were excluded as described previously.^25^

### 2.3 Data processing and registration

#### 2.3.1 Gray matter

T1‐weighted images were used to derive parcellated GM regions of interest (ROIs). The FreeSurfer recon‐all pipeline^27^ was applied for intensity normalization, skull stripping, subcortical segmentation, and parcellation according to ENIGMA guidelines. All FreeSurfer surfaces were visually inspected and manually corrected as needed.^24^

#### 2.3.2 Superficial WM

Diffusion‐weighted MRI data from both cohorts underwent the same preprocessing steps: denoising,^28^ Gibbs unringing,^29^ and signal drift correction.^30^ Because one cohort lacked reverse phase‐encoded B0 images, Synb0‐DisCo^31^, ^32^ was used to generate synthetic undistorted B0s from T1‐weighted images for all participants to ensure consistency. These synthetic images were bias‐corrected using N4,^33^ followed by distortion correction with TOPUP^34^, ^35^ and EDDY^36^ to address susceptibility warping, eddy currents, and motion. Tensor maps were then computed using FSL's DTIFIT.^35^ Mean diffusivity (MD) maps were registered to standard space using both affine and nonlinear (symmetric diffeomorphic) registration via ANTs,^37^ aligned to the FSL “FMRIB158_2mm” MD template.^35^ The resulting linear and nonlinear transformations were applied to all tensor maps using trilinear interpolation. For SWM analysis, only WM voxels within 5 mm of the GM–WM boundary in defined ROIs were included, based on prior work indicating that this range captures maximal differences from controls.^12^

#### 2.3.3 Intracranial EEG

We followed the preprocessing steps from Woodhouse et al.^25^ RAM and UCLH recordings were downsampled to 200 Hz, and a common average reference montage was applied. In the RAM cohort, channels within the SOZ, early propagation zones, lesions, or WM were excluded, yielding 21 927 channels across 247 participants. Seventy seconds of awake interictal iEEG at least 2 h postseizure were extracted, as supported by prior studies^21^, ^38^, ^39^ showing that this duration is representative of longer recordings. Only GM channels without artifacts were included, resulting in 2974 channels from 40 patients.

**FIGURE 1:**
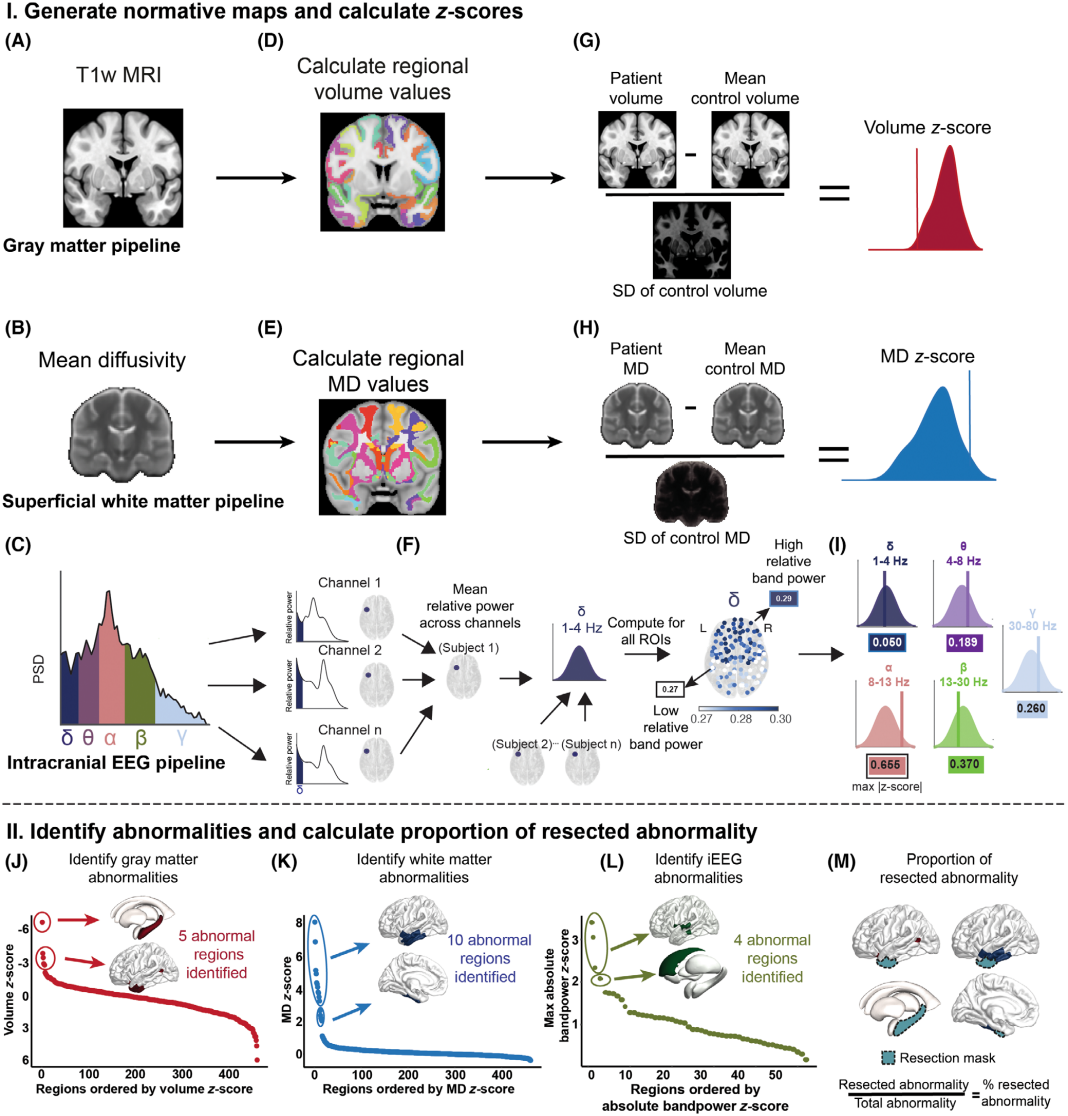
I. Generate normative maps and compute *z*‐scores. (A, B) Individual T1‐weighted (T1w) magnetic resonance imaging (MRI) and mean diffusivity (MD) maps provide volume and superficial white matter MD values. (C) Intracranial electroencephalographic (iEEG) band power was calculated across five frequency bands (δ, 1–4 Hz; θ, 4–8 Hz; α, 8–13 Hz; β, 13–30 Hz; γ, 30–80 Hz). (D, E) Regional volume and MD values were extracted, and (F) mean relative band power was averaged across electrodes within each region of interest (ROI; example shown for δ band). (G–I) All values were *z*‐scored relative to the normative maps to quantify deviation from the healthy mean. II. Identify abnormalities and calculate resection overlap. (J–L) *Z*‐scores were ranked per modality, and abnormal ROIs were identified using change point analysis. (M) For each subject, abnormal regions were intersected with the resection mask to compute the proportion of abnormal ROIs that were resected. Methods were extended from Kozma et al.^23^ PSD, power spectral density.

Based on the preprocessed iEEG time series, we estimated the full power spectrum for each 70‐s epoch. Using Welch's method, the data were segmented into 2‐s windows with 1‐s overlap, a Hamming window was applied, and the Fourier transform was computed and averaged across segments. We calculated the band power within five frequency bands of interest (δ, 1–4 Hz; θ, 4–8 Hz; α, 8–13 Hz; β, 13–30 Hz; and γ, 30–80 Hz), as this approach has shown promising results in previous studies.^19^, ^21^ The values were then log_10_ transformed and normalized, so that the sum of the band power in each contact was equal to 1 (Figure 1C).

### 2.4 Abnormality calculation

The Lausanne parcellation^40^ was used, comprising 446 neocortical and 14 deep brain regions, including the hippocampus, amygdala, thalamus, putamen, and caudate.

#### 2.4.1 GM and SWM

We used the healthy controls to compute normative baselines of GM volume and adjacent SWM MD per region. We selected MD in addition due to its value in abnormality localization.^13^, ^15^ MD reflects average diffusion in all directions, with higher values indicating more unrestricted diffusion, often linked to myelin disruption and increased extracellular space in WM,^41^, ^42^ which are common in epilepsy.^10^ For the SWM, we took the mean MD of WM voxels within 5 mm of the nearest region in the Lausanne parcellation. We calculated regional means and SDs, harmonizing across scanning protocols using ComBat^43^ and adjusting for covariates (age and sex). For individuals with TLE, we calculated abnormalities by *z*‐scoring each region's MD and volume values against the corresponding normative map. These abnormalities quantified deviations from the healthy mean in each region for both modalities (Figure 1A,B,D,E,G,H). We focused on negative *z*‐scores for GM abnormalities, reflecting volume reductions. For SWM, we analyzed positive *z*‐scores in MD, which indicate increased diffusion.^4^, ^10^

#### 2.4.2 Intracranial EEG

To assess the abnormality of a region's relative band power in a patient, relative to the normative map, we calculated the absolute *z*‐score of each region in each frequency band. We used the maximum absolute *z*‐score across frequency bands to define abnormality at the regional level, considering individual differences in frequency bands (Figure 1F,I).

#### 2.4.3 Identifying abnormal regions

For each patient, regional abnormalities were ranked separately within each modality. GM regions were ranked from most negative to most positive values (reflecting expected volume loss as abnormal), whereas SWM and iEEG rankings were reversed (as increased MD and absolute band power indicate abnormality). Multiple change point (MCP) analysis^44^ was then used to determine patient‐specific thresholds to identify abnormal ROIs (Figure 1J,L). MCP applies Bayesian regression to detect shifts in mean, variance, or autocorrelation; here, we used it to identify mean shifts as thresholds distinguishing abnormal regions from the rest of the distribution. We evaluated the spatial distribution of MRI (union of GM and SWM) as well as MRI‐iEEG (union of GM, SWM, and iEEG) abnormalities. We combined GM, SWM, and iEEG abnormalities using their union, which includes all regions of interest identified as abnormal in either or all the modalities, as this approach has already shown value for abnormality localization.^23^ We also considered using the concordance of modalities; however, this was rare and excluded most data, making it an overly restrictive approach.^23^

### 2.5 Resection mask generation

Resection masks were created using a semiautomated pipeline,^45^ based on postoperative imaging aligned to preoperative space. The initial masks were generated using FastSurfer,^46^ ANTs,^37^ and ATROPOS,^47^ then visually inspected and manually corrected as needed.^45^ All patients underwent the same surgical approach, with larger resections performed on the non‐language‐dominant hemisphere (typically the right). Finalized resection masks were registered to MNI‐152 standard space to match the abnormality maps. Similarly to previous work, ROIs with more than 10% overlap with the resection mask were considered resected.^21^, ^23^, ^38^

For each subject, abnormality maps were overlaid with the resection mask to assess spatial overlap between resected tissue and abnormal ROIs (Figure 1M). We calculated the proportion of abnormal ROIs that were resected and compared this between ILAE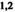 and ILAE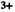 outcome groups.

### 2.6 Statistical analysis

We compared the proportion of resected MRI as well as combined MRI and iEEG abnormalities between patients who became free of disabling seizures (ILAE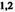) and those who did not (ILAE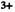). Group differences were quantified using the area under the receiver operating characteristic curve (AUC) as well as precision–recall performance (area under the precision–recall curve [AUPRC]). Statistical significance was assessed using a one‐tailed Wilcoxon rank‐sum test to test the hypothesis that individuals free of disabling seizures would have a higher proportion of resected abnormalities. To evaluate the likelihood of an ROI being iEEG‐implanted or abnormal while being an MRI abnormality, we used a binomial logistic regression model. Finally, to assess whether combining interictal iEEG abnormalities with MRI abnormalities significantly improved outcome differentiation, we applied DeLong test for correlated ROC curves, which compares AUCs from paired differentiations. Finally, to address the potential presence of overfitting, we have conducted a leave‐one‐out cross‐validation analysis (see Supplementary S6).

### 2.7 Code and data availability

Code used to produce the figures is available at the following location: https://github.com/cnnp‐lab/three‐modality.

## 3 RESULTS

### 3.1 Resection of MRI abnormalities differentiates outcome in complex cases

We investigated the location and resection of MRI abnormalities. Regions were defined as MRI abnormal if either the GM volume or SWM MD (or both, i.e., their union) was abnormal using the change point analysis.

**FIGURE 2:**
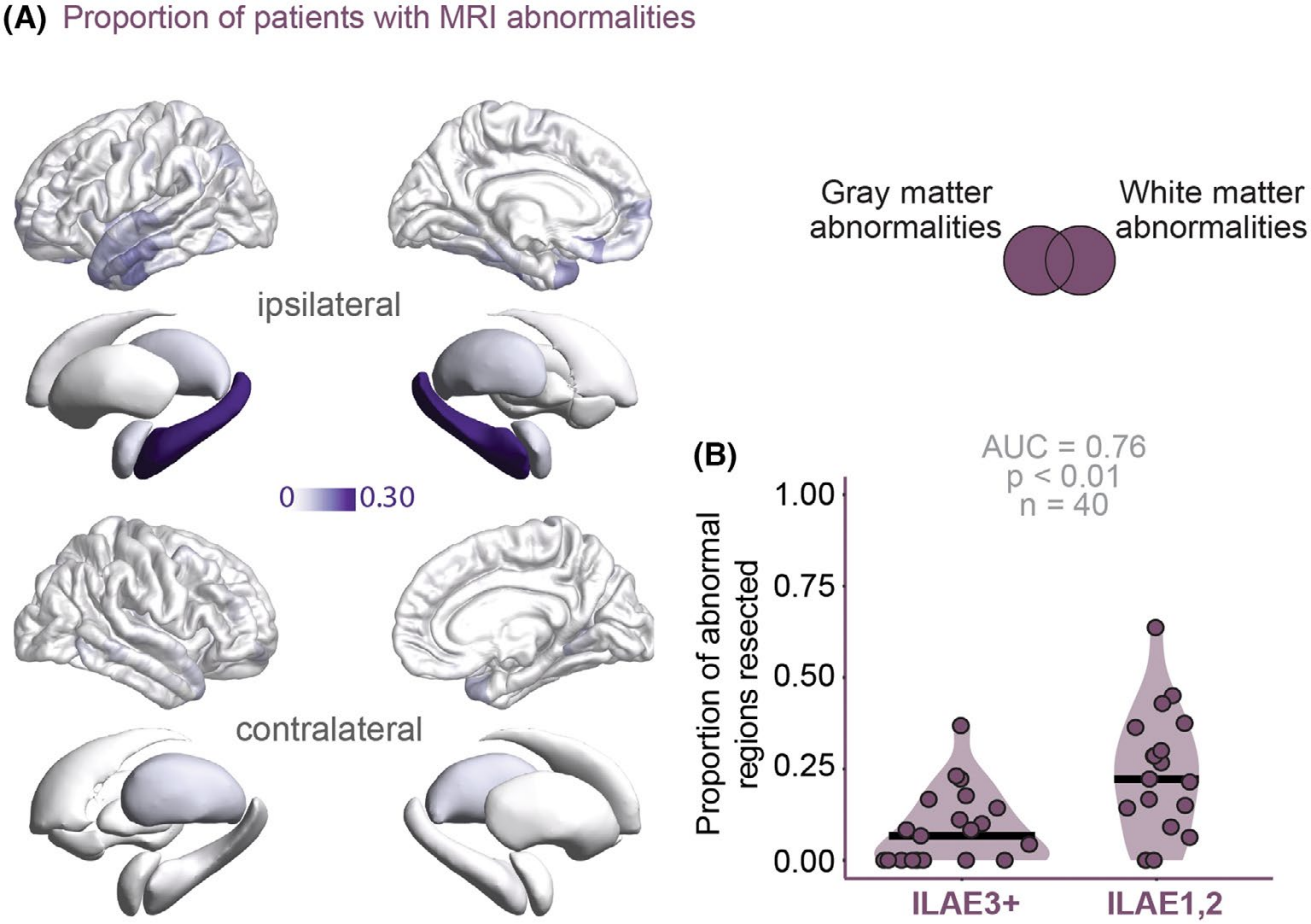
Resecting magnetic resonance imaging (MRI) abnormal regions differentiates postsurgical outcomes based on their spatial distribution. (A) Spatial distribution of the MRI abnormalities across the cohort are prevalent in the temporal lobe. (B) Resection of a higher proportion of abnormalities is associated with seizure freedom postsurgically (International League Against Epilepsy [ILAE] class 1 and 2). Each point represents a patient; the darker lines show the median. AUC, area under the curve.

Across the cohort, the ipsilateral hippocampus was abnormal in 30% of patients, and regions in the inferior temporal lobe were abnormal in 10%–20% patients; there were minimal abnormalities elsewhere (Figure 2A). Patients with a greater proportion of abnormal regions resected were significantly more likely to be free from disabling seizures (AUC = .76, AUPRC = .78, *p* < .01; Figure 2B).

### 3.2 MRI abnormalities overlap with iEEG implantation in individuals free from disabling seizures

**FIGURE 3:**
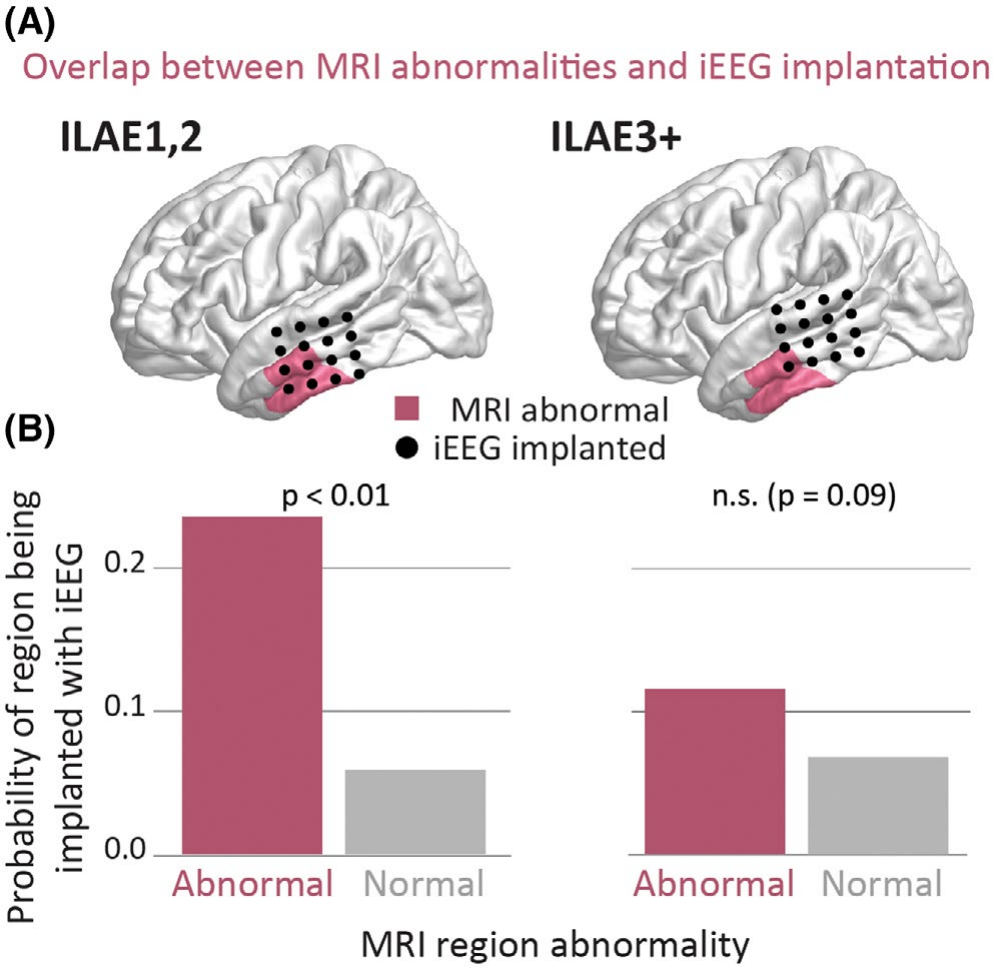
Magnetic resonance imaging (MRI) abnormal regions are likely to be implanted but differ by outcome. (A) Illustrative patient example showing MRI abnormalities and intracranial electroencephalographic (iEEG) implantation overlap. (B) Probability of iEEG implantation based on MRI abnormalities, stratified by outcome group. For International League Against Epilepsy (ILAE) class 1 and 2 patients, a significant effect is present (*p* < .01), in contrast to the ILAE3+ patients (*p* = .09). n.s., not significant.

MRI abnormal regions were more likely to be implanted compared to those not identified as abnormal in patients with ILAE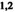 outcomes (*z* = 6.26, *p* < .01; Figure 3B). However, no significant difference in implantation likelihood was observed for patients with ILAE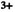 outcomes (*z* = 2.23, *p* = .09; Figure 3B).

### 3.3 MRI and iEEG abnormalities overlap in individuals free from disabling seizures

**FIGURE 4:**
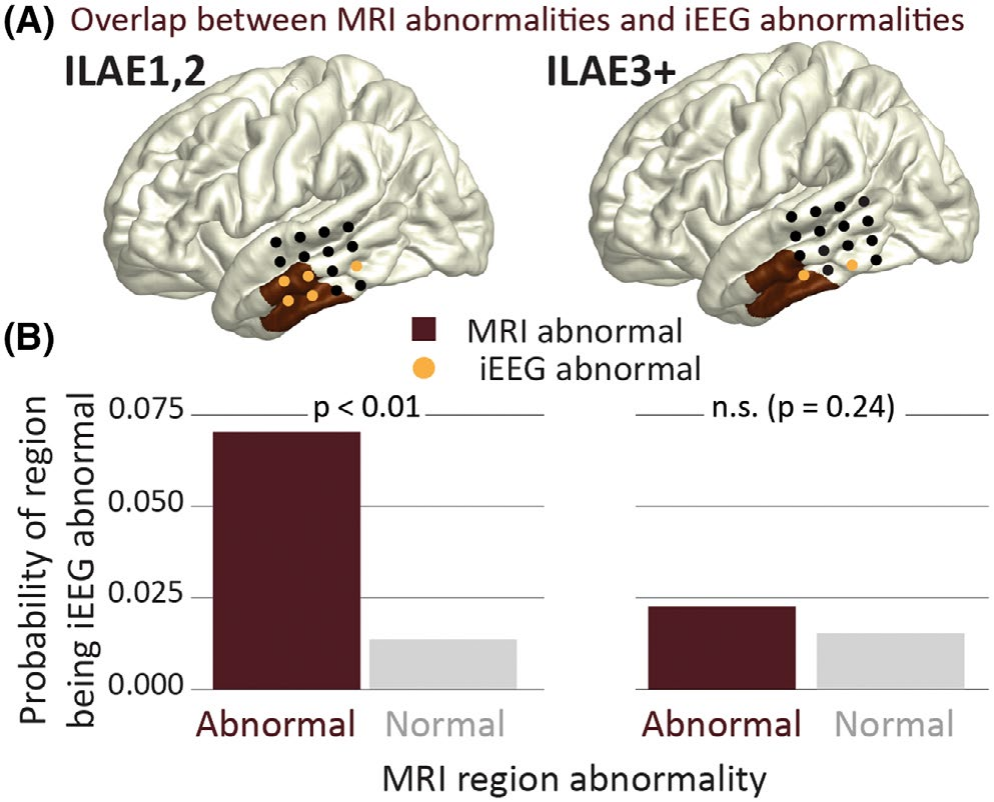
Magnetic resonance imaging (MRI) abnormal regions are likely to be intracranial electroencephalographic (iEEG) abnormal but differ by outcome. (A) Individual patient example showing MRI abnormalities and iEEG abnormality overlap. (B) Probability of iEEG abnormality based on MRI abnormalities, stratified by outcome group. ILAE, International League Against Epilepsy; n.s., not significant.

MRI abnormal ROIs were significantly more likely to also have iEEG abnormalities in individuals with favorable outcomes (Figure 4B; ILAE_1,2_; *z* = 4.34, *p* < .01). This pattern was not observed in patients with poorer outcomes (ILAE_3+_; *z* = .81, *p* = .24).

### 3.4 Integration of MRI and iEEG abnormalities significantly improves outcome differentiation

**FIGURE 5:**
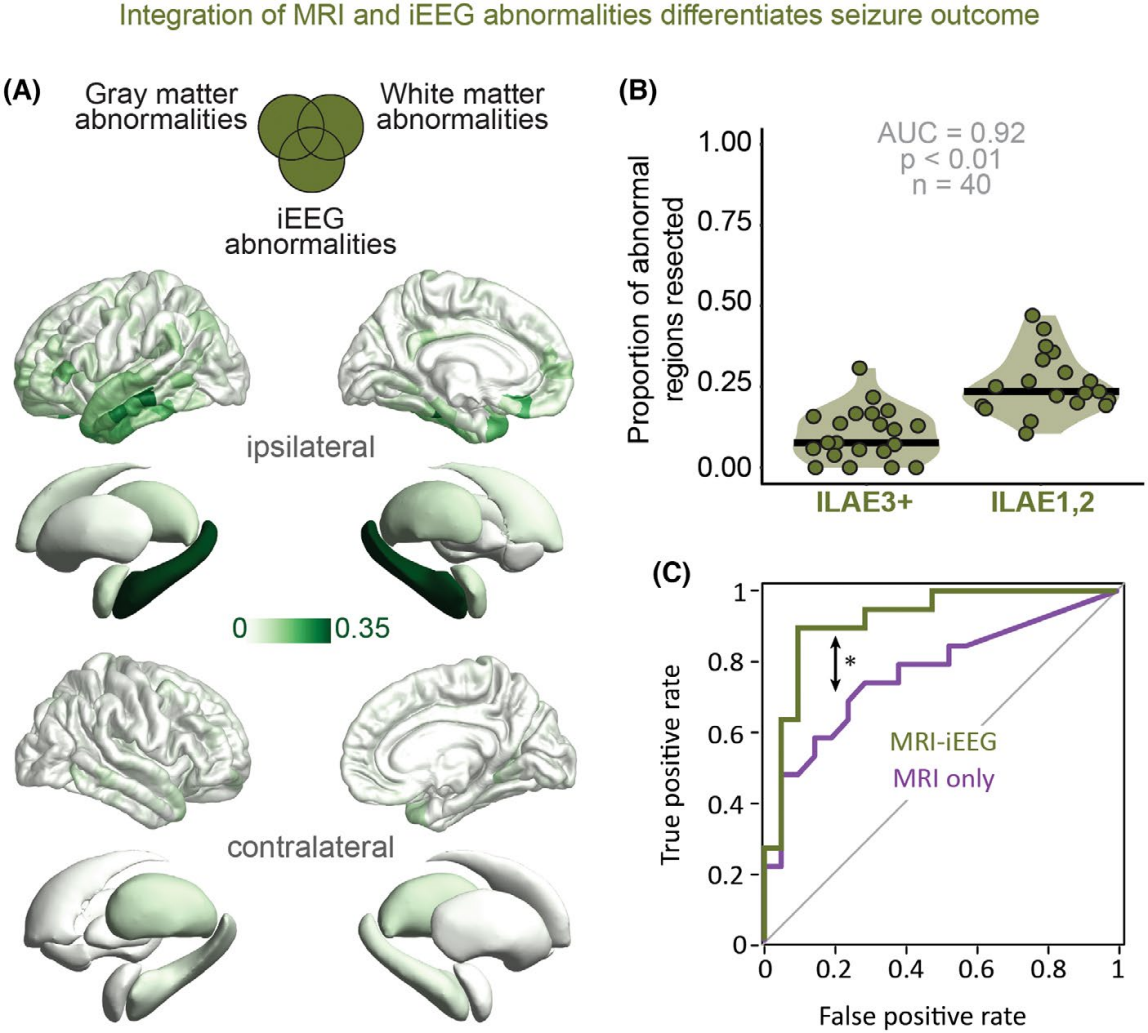
Resecting the combination of magnetic resonance imaging (MRI) and intracranial electroencephalographic (iEEG) abnormal regions improves outcome differentiation compared to the resection of only MRI abnormal regions. (A) Spatial distribution of the MRI‐iEEG abnormalities. (B) Proportion of resected abnormal regions distinguishes patients without disabling seizures. Each point represents a patient, with the darker lines indicating the median. (C) Receiver operating characteristic curve comparing the MRI only and MRI‐iEEG abnormalities in differentiating surgical outcomes. *Indicates significant improvement in outcome differentiations. AUC, area under the curve; ILAE, International League Against Epilepsy.

Regions that were MRI and/or iEEG abnormal were most common in the ipsilateral hippocampus (35%) and the anterior portion of the temporal lobe (Figure 5A). Resecting the combination of interictal iEEG abnormalities and MRI abnormalities yielded a significant increase (*z* = 2.01, *p* < .05; Figure 5C) in outcome differentiation (AUC = .92, AUPRC = .89, *p* < .01; Figure 5B) compared to the resection of MRI abnormalities alone.

Our findings were consistent across different acquisition protocols and in individuals with HS, other pathologies, and unremarkable MRI (Figures S2–S4). The overlap between MRI abnormalities and iEEG implantation or abnormalities was not driven by any single individual (Figure S5). Using SOZs or interictal spikes instead of interictal band power did not improve further outcome differentiation (Figure S1). To evaluate the relationship between electrode coverage and surgical outcomes, we assessed bilateral iEEG sampling (Table S7). Finally, for all patients, we calculated the percentage of the putative location of residual seizure focus, which means the ROIs that are believed (based on current evidence such as iEEG and MRI) to still contain epileptogenic tissue after prior resection. We categorized as ipsilateral periresection cortex, contralateral corresponding cortex, or indeterminate. A detailed patient‐level summary table and corresponding figure are provided in Data S1: Section 8.

## 4 DISCUSSION

In this study, we demonstrate that resection of structural MRI abnormalities reliably differentiates surgical outcomes, including in those requiring iEEG, supporting the broader use of MRI‐based biomarkers. Structurally abnormal regions were more likely to be implanted and showed greater alignment with iEEG abnormalities. When these regions were resected, patients tended to have better outcomes. We also found that integrating MRI and interictal iEEG abnormalities significantly improves outcome differentiation. These findings support a multimodal, data‐driven approach to guide implantation and optimize resection.

Our results show that resection of structural MRI abnormalities differentiates surgical outcomes in TLE, including in complex cases requiring iEEG. This validates our previous approach,^23^ which is particularly important as iEEG patients typically have worse outcomes due to challenges in EZ localization.^3^ In this subset, 47% achieved freedom from disabling seizures of those who were MRI‐negative, compared to 73% in the broader cohort.^24^ Abnormalities were most commonly found in the ipsilateral hippocampus and temporal cortical regions, consistent with prior reports of GM^4^, ^48^ and SWM abnormalities^8^, ^12^, ^49^ in TLE.

To detect epileptogenic abnormalities, iEEG electrode placements are designed to delineate the EZ. As expected, we found that structural abnormalities were much more likely to be implanted in subjects who became free from disabling seizures (ILAE_1,2_, *p* < .01). However, in individuals with ILAE_3+_ outcomes, we observed more frequent mismatches between MRI abnormalities and implanted regions (*p* = .09). These mismatches could contribute to incomplete resections and, in turn, poorer outcomes.^50^, ^51^ Our findings support a two‐step strategy: (1) quantitatively using structural abnormalities to guide implantation and (2) leveraging functional data from iEEG to refine localization.

Clinically, multiple modalities are integrated to localize the suspected EZ. Quantitative multimodal approaches improve localization compared to unimodal methods.^8^, ^52^, ^53^, ^54^ Adding a third modality (iEEG) enhanced differentiation of surgical outcomes, likely by capturing functional abnormalities alongside structural ones. Different modalities may detect distinct aspects of pathology, which is important given the heterogeneity of focal epilepsy, even within TLE.^55^, ^56^ Multimodal strategies may increase sensitivity across diverse patient populations where underlying pathology is variable or unknown. This aligns with prior studies showing that single modality analyses underperform in MRI‐negative cases, likely due to more diffuse or heterogeneous abnormalities.^23^, ^57^, ^58^, ^59^ Similarly, in those without HS, MRI alone yielded only modest performance in outcome differentiation, whereas the MRI‐iEEG approach maintained strong performance.

We used changes in interictal band power to identify functional abnormalities, as prior studies have shown their relevance for EZ localization and outcome delineation.^19^, ^21^, ^22^, ^38^ However, traditional biomarkers such as SOZ and interictal spikes did not improve further outcome differentiation in our cohort. The SOZ may be only part of the EZ, and seizure onsets can be inconsistent or diffuse.^60^, ^61^, ^62^ Similarly, interictal spikes are commonly observed in epilepsy and have been proposed as biomarkers of epileptogenic tissue.^63^, ^64^, ^65^, ^66^ However, their surgical removal does not always reliably differentiate outcomes.^21^ Whereas some studies suggest spikes may outperform other features in guiding temporal lobe surgery,^63^, ^67^ others report no clear advantage of them.^68^, ^69^ In our cohort, band power abnormalities outperformed both SOZ and spikes as functional markers for surgical planning.

This study has some limitations. First, we assumed all structural and functional abnormalities were potentially epileptogenic, although some may have been incidental or seizure‐induced rather than causal.^70^, ^71^ Second, we focused exclusively on TLE to reduce heterogeneity; future work should extend these methods to extratemporal epilepsy.^6^, ^8^, ^49^, ^53^ Third, structural abnormalities were assessed using GM volumetry and SWM MD, which has shown sensitivity in voxelwise EZ analyses.^13^, ^15^ However, additional metrics such as cortical thickness^72^ and fractional anisotropy in SWM^10^, ^12^, ^49^, ^52^, ^73^ may offer complementary information, especially for subtle or extratemporal abnormalities. Finally, the sample size was modest (*N* = 40) and should be validated in larger cohorts.

To summarize, this study shows that quantitatively combining MRI‐detected GM and SWM abnormalities with interictal iEEG features improved outcome differentiation in TLE. Individuals with a higher proportion of resected multimodal abnormalities were more likely to be free from disabling seizures. These findings support a multimodal, data‐driven approach to enhance localization accuracy and guide more effective surgical planning.

## AUTHOR CONTRIBUTIONS

**Peter N. Taylor:** Writing—review & editing; supervision; conceptualization; funding acquisition. **Yujiang Wang:** Resources; conceptualization. **Beate Diehl:** Resources. **Andrew W. McEvoy:** Resources. **Gerard Hall:** Resources; conceptualization. **Callum Simpson:** Resources. **Jane de Tisi:** Resources. **John S. Duncan:** Resources; conceptualization; writing—review & editing. **Sjoerd B. Vos:** Writing—review & editing; data curation; resources. **Gavin P. Winston:** Writing—review & editing; investigation; data curation; resources; conceptualization; funding acquisition. **Csaba Kozma:** Writing—review & editing; writing—original draft; visualization; methodology; formal analysis; data curation; conceptualization. **Jonathan Horsley:** Supervision; conceptualization; resources. **Anna Miserocchi:** Resources.

## FUNDING INFORMATION

C.K. is supported by Epilepsy Research Institute UK. P.N.T. and Y.W. are both supported by UKRI Future Leaders Fellowships (MR/T04294X/1, MR/V026569/1). G.P.W. and scan acquisition were supported by the MRC (G0802012, MR/M00841X/1). J.S.D. and J.d.T. are supported by the NIHR UCLH/UCL Biomedical Research Centre.

## CONFLICT OF INTEREST STATEMENT

None of the authors has any conflict of interest to disclose. We confirm that we have read the Journal's position on issues involved in ethical publication and affirm that this report is consistent with these guidelines.

## Supporting information


DATA S1

## Data Availability

The data that support the findings of this study are available on request from the corresponding author. The data are not publicly available due to privacy or ethical restrictions.
