# Supplementary material for: Multimodal integration of magnetic resonance imaging and intracranial electroencephalographic abnormalities in temporal lobe epilepsy surgery
Source: Epilepsia. 2025 Dec 22;67(3):1181–92. doi: 10.1111/epi.70042 (PMC13007827; doi:10.1111/epi.70042)
Supplement: Supplementary file 1 — DATA S1 [file EPI-67-1181-s001.docx]

Multimodal integration of MRI and iEEG abnormalities in temporal lobe epilepsy surgery

Csaba Kozma${}^{1*}$, Jonathan Horsley${}^{1}$, Gerard Hall${}^{1}$, Callum Simpson${}^{1}$, Jane de Tisi${}^{3}$,
Anna Miserocchi${}^{3}$, Beate Diehl${}^{3}$, Andrew W. McEvoy${}^{3}$, Sjoerd B. Vos${}^{3,4,5}$,
Gavin P. Winston${}^{3,6}$, Yujiang Wang${}^{1,2,3}$, John S. Duncan${}^{3}$, Peter N. Taylor${}^{1,2,3}$

1. CNNP Lab (www.cnnp-lab.com), School of Computing, Newcastle University, Newcastle upon Tyne, United Kingdom
2. Translational and Clinical Research Institute, Faculty of Medical Sciences, Newcastle University, Newcastle upon Tyne, United Kingdom
3. Department of Epilepsy, UCL Queen Square Institute of Neurology, University College London, London, United Kingdom
4. Western Australia National Imaging Facility, The University of Western Australia, Nedlands, Australia
5. Centre for Medical Image Computing, Computer Science Department, University College London, London, United Kingdom
6. Department of Medicine, Queen’s University, Kingston, Ontario, Canada

* c.a.kozma2@newcastle.ac.uk, peter.taylor@newcastle.ac.uk

HIGHLIGHTS:

- Resection of gray and superficial white matter abnormalities together reliably differentiated surgical outcomes, even in complex cases.
- Combining MRI and interictal iEEG abnormalities significantly improved outcome differentiation.
- In cases free from disabling seizures, regions with abnormal gray and superficial white matter were likely to be implanted and resected.

KEYWORDS: gray matter, superficial white matter, iEEG, multimodal, epilepsy surgery

We confirm that we have read the Journal’s position on issues involved in ethical publication and affirm that this report is consistent with these guidelines. Supporting information is included in a separate file.

# Supplementary

# Delineation of SOZ or interictal spiking does not improve outcome differentiation


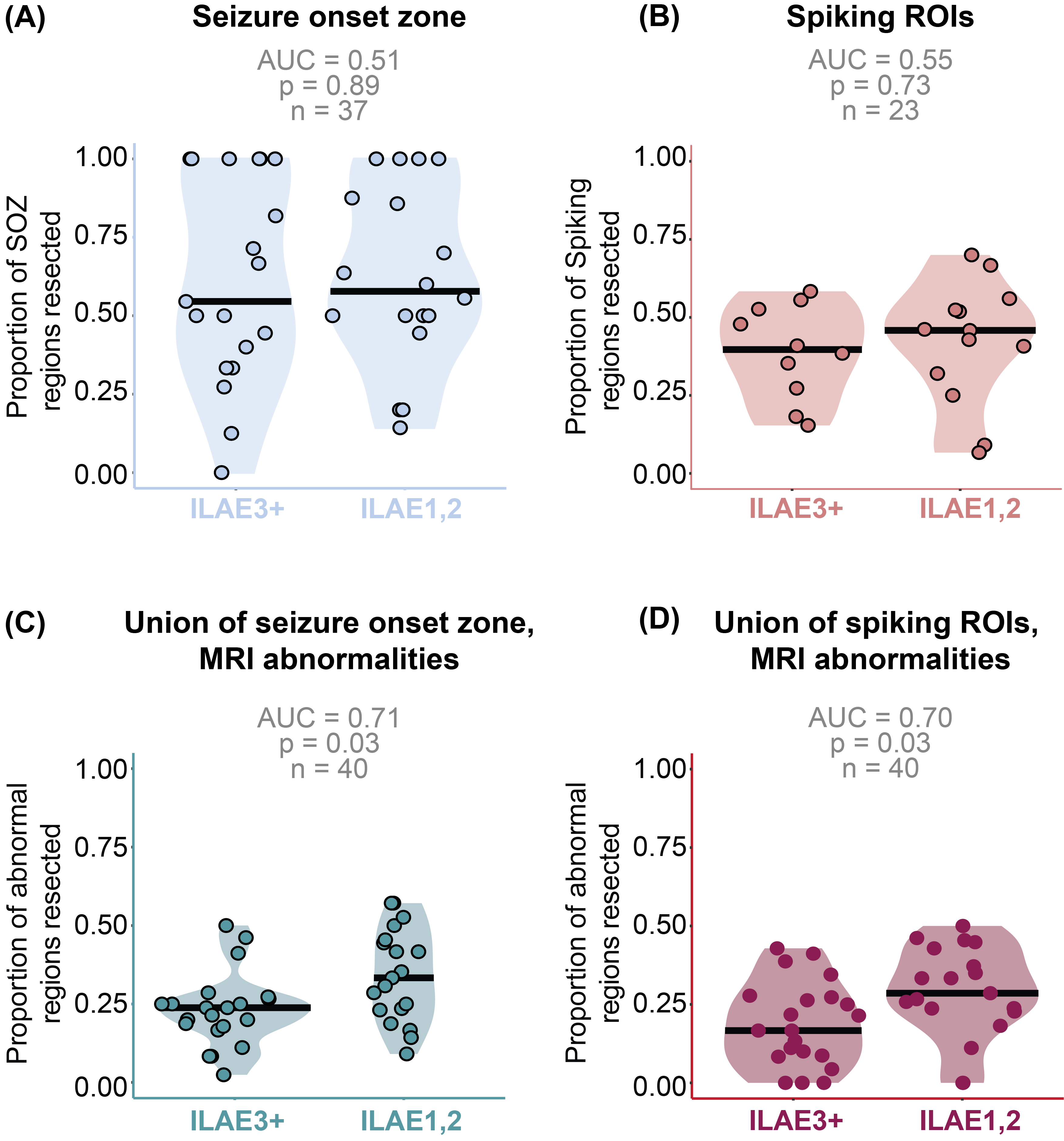


**Figure S1: Resecting seizure onset zone or spiking regions does not differentiate better post-surgical outcome:** Comparison of resected (A) seizure onset zone, (B) spiking ROIs, (C) MRI abnormality and seizure onset zone, (D) MRI abnormality and spiking ROIs between ILAE${}_{1,2}$ and ILAE${}_{3+}$ patients. Each point represents a patient, with the darker line indicating the median.

Resecting regions identified as seizure onset zones (SOZ) (AUC = 0.51, AUPRC = 0.49, p = 0.89; Fig. S1A) or those with spiking activity (AUC = 0.55, AUPRC = 0.52, p = 0.73; Fig. S1B) did not significantly improve differentiation between surgical outcomes. Even when combining gray matter, superficial white matter, and SOZ (AUC = 0.71, AUPRC = 0.70, p = 0.03; Fig. S1C) or spiking ROIs (AUC = 0.70, AUPRC = 0.70, p = 0.03; Fig. S1D), these features did not enhance outcome differentiation compared to using interictal iEEG bandpower abnormality. Although interictal spikes are commonly used as clinical markers and are frequently observed in EEGs, analysis in Fig. S1 shows that resecting spiking regions does not increase accuracy of distinguishing outcome groups. Furthermore, if spikes were the primary source of abnormality, they would substantially alter the maximum absolute z-score, which we did not observe.

# Results of outcome differentiation hold in both acquisition cohorts


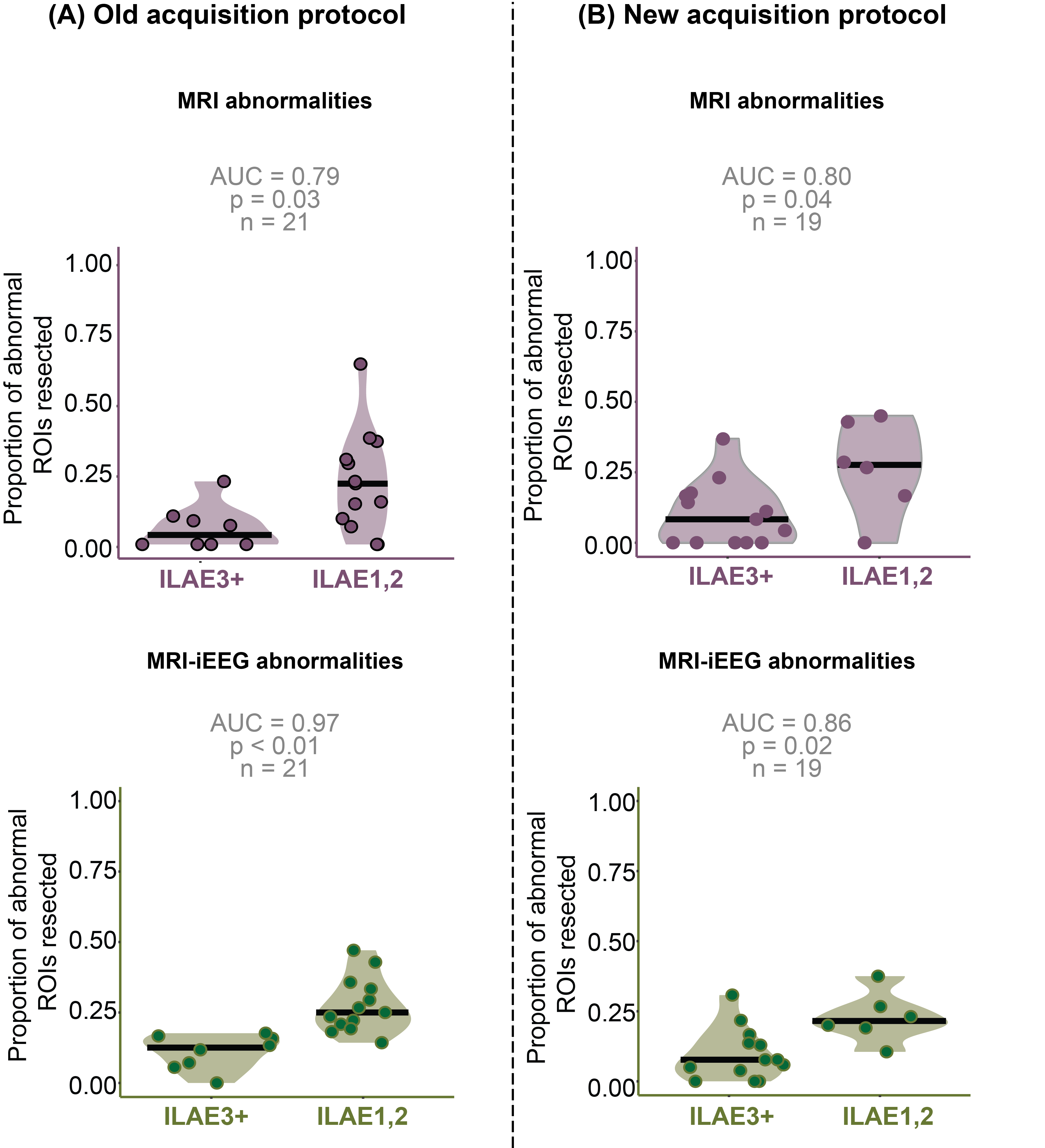


**Figure S2: Replication of surgical outcome findings in two acquisition cohorts using GM, SWM, iEEG and their combinations:** Comparison of resected abnormal ROIs in ILAE${}_{1,2}$ vs ILAE${}_{3+}$ patients using (A) old and (B) new imaging protocols across MRI and MRI-iEEG metrics. Each point represents a patient, with a darker line marking the median.

Data were collected using two imaging protocols. The first cohort (87 patients, 29 controls) was scanned from 2009–2013 on a 3T GE Signa HDx scanner with T1-weighted images (1.1 mm slices) and DWI (52 directions, b = 1,200 s/mm²). The second cohort (56 patients, 67 controls) was scanned from 2014–2019 on a 3T GE MR750 scanner with improved gradients, T1-weighted images (1 mm slices), and DWI comprising 115 volumes across four b-values.

In both cohorts, the resection of MRI and MRI-iEEG abnormalities effectively differentiated ILAE${}_{1,2}$ vs. ILAE${}_{3+}$ outcomes (old cohort: MRI abnormalities AUC = 0.79, AUPRC = 0.88, p = 0.03; MRI-iEEG abnormalities AUC = 0.97, AUPRC = 0.98, p $<$ 0.01; new cohort: MRI abnormalities AUC = 0.80, AUPRC = 0.73, p = 0.04; MRI-iEEG abnormalities AUC = 0.86, AUPRC = 0.69, p $<$ 0.01; Fig. S2). While the newer protocol improved performance in the MRI abnormalities, it slightly underperformed in the MRI-iEEG abnormalities compared to the older protocol.

# MRI negative patients


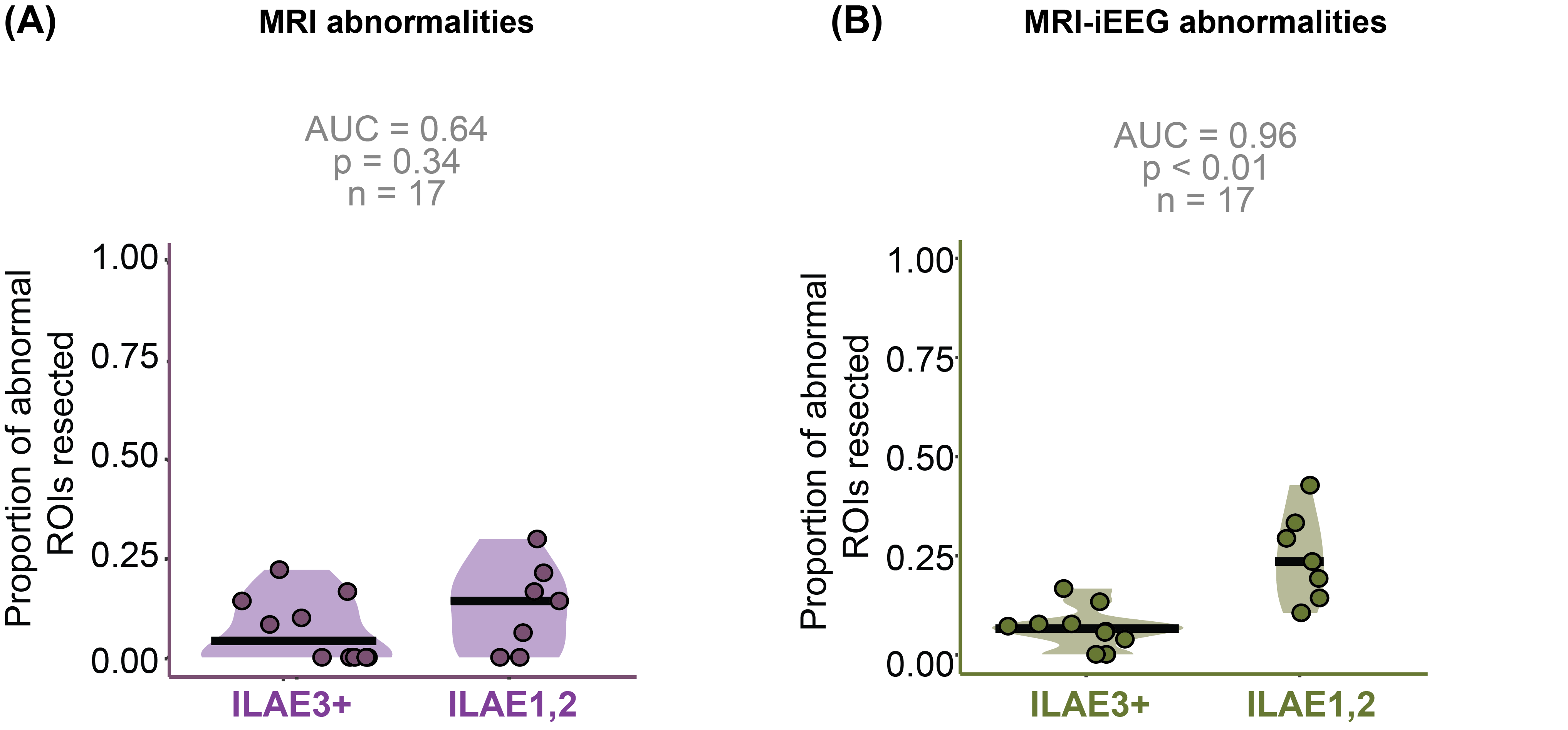


**Figure S3: Replication of surgical outcome findings in MRI negative individuals using GM, SWM, iEEG and their combinations:** Comparison of resected abnormal ROIs between ILAE${}_{1,2}$ and ILAE${}_{3+}$ patients across (A) MRI , (B) MRI-iEEG. Each point represents a patient, with the darker line indicating the median.

We replicated our analysis in MRI-negative individuals. Among the 40 patients with TLE, only 17 were MRI-negative, limiting the scope of subgroup analysis. Nevertheless, both MRI only and MRI-iEEG approaches effectively differentiated ILAE${}_{1,2}$ and ILAE${}_{3+}$ outcomes in this group (MRI abnormalities AUC = 0.64, AUPRC = 0.59, p = 0.34; MRI-iEEG abnormalities AUC = 0.96, AUPRC = 0.94, p $<$ 0.01; Figure S3). However, MRI only approach underperformed compared to prior findings^23^. MRI-iEEG results highlight the need for replication in larger MRI-negative cohorts.

# Abnormalities in non-HS vs HS


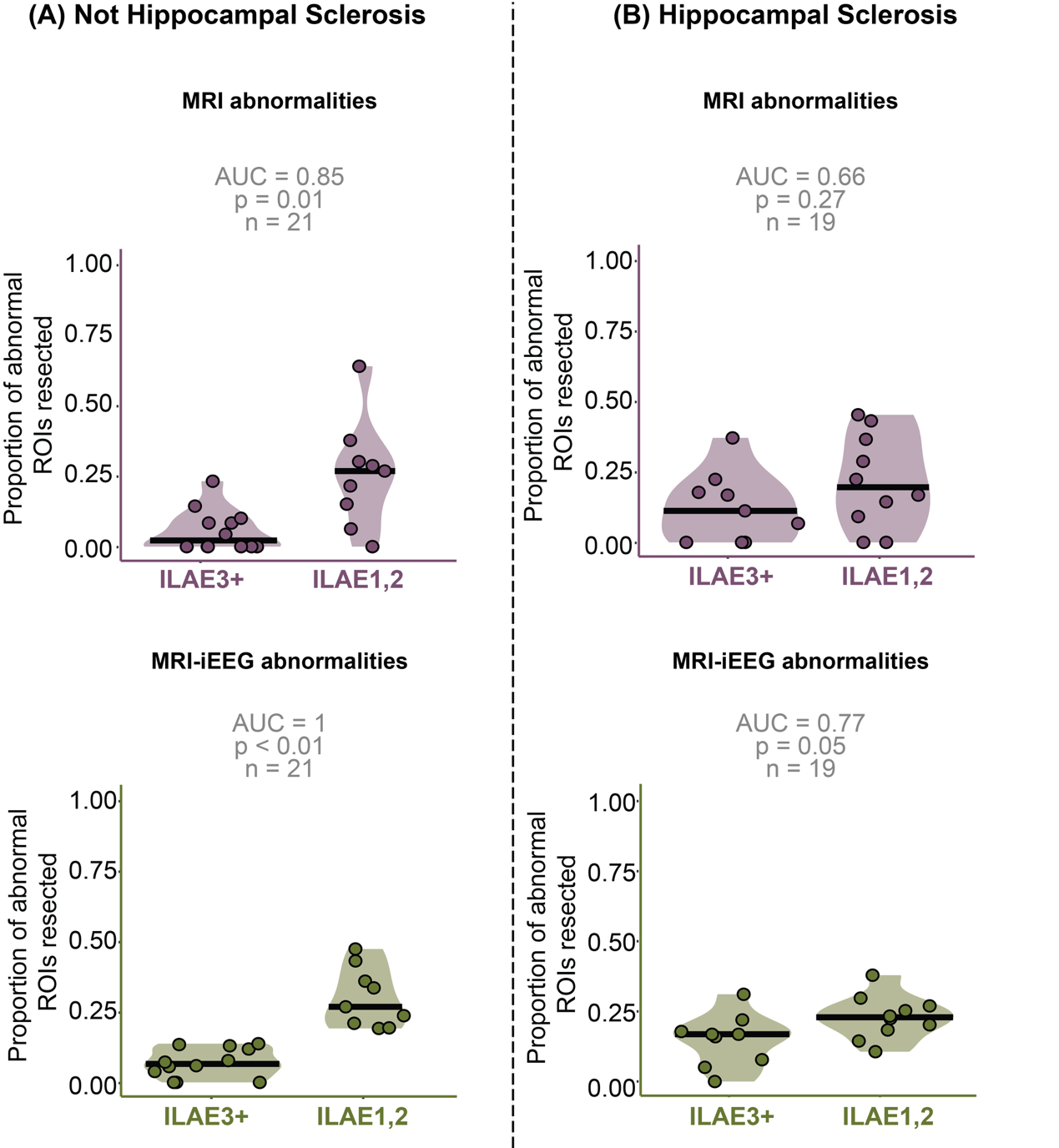


**Figure S4: Replication of surgical outcome findings in HS and non-HS patients using MRI only and MRI-iEEG approaches:** Comparison of resected abnormal ROIs in ILAE${}_{1,2}$ vs ILAE${}_{3+}$ patients splitting to (A) non-HS and (B) HS cases across MRI only and MRI-iEEG approaches. Each point represents a patient, with a darker line marking the median.

Results were consistent across both HS and non-HS cases when using the MRI-iEEG approach. However, in non-HS cases, the MRI abnormalities alone underperformed (non-HS AUC = 0.85, AUPRC = 0.86, $p<0.01$; HS AUC = 0.66, AUPRC = 0.72, $p=0.27$; Fig. S4). In contrast, MRI-iEEG showed strong performance in both groups (non-HS AUC = 1.00, AUPRC = 1.00, $p<0.01$; HS AUC = 0.77, AUPRC = 0.75, $p=0.05$), though these results should be interpreted cautiously due to the limited number of concordant cases.

MRI only and MRI-iEEG abnormalities better differentiate outcomes in HS cases aligns with prior studies linking HS to cortical thinning and more localized, MRI-visible pathology^4,23,74^, likely reflecting a more focal disease process that is easier to target surgically. Conversely, the weaker performance of structural markers in non-HS cases suggests more diffuse or heterogeneous epileptogenic networks^75–77^. The improved performance of the MRI-iEEG approach in non-HS cases underscores the importance of multimodal approaches in this subgroup.

# MRI abnormalities overlap with iEEG implantation and abnormality in individuals free from disabling seizures


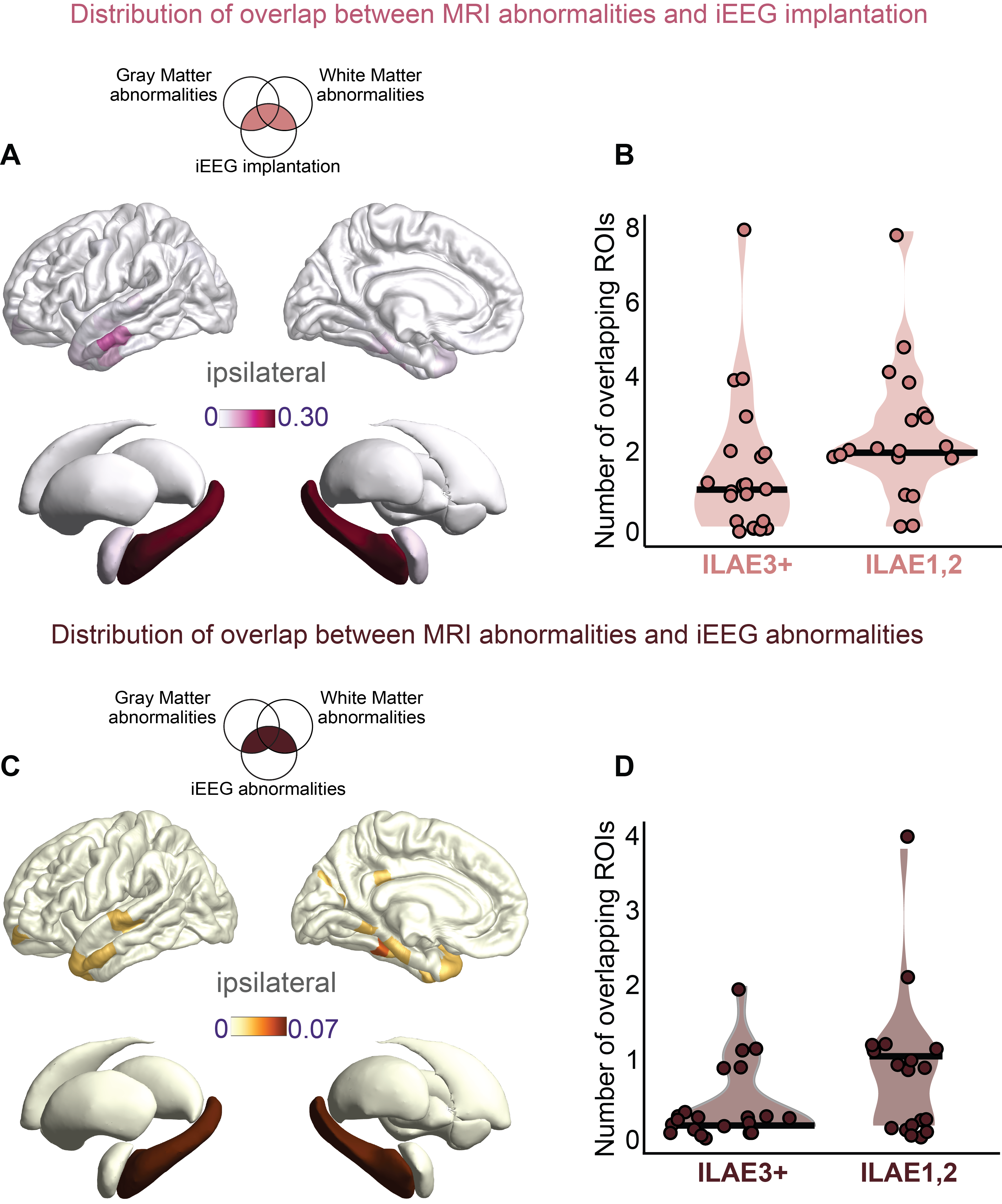


**Figure S5: Overlapping ROIs between MRI abnormalities and iEEG implantation and abnormality**: (A) Spatial distribution of the overlap between MRI abnormalities and iEEG implantation. (B) Comparison of the overlapping MRI abnormal and iEEG implanted ROIs in ILAE${}_{1,2}$ vs ILAE${}_{3+}$ patients. (C) Spatial distribution of the overlap between MRI abnormalities and iEEG abnormality. (D) Comparison of the overlapping MRI and iEEG abnormal ROIs in ILAE${}_{1,2}$ vs ILAE${}_{3+}$ patients. In both (B) and (D), each point represents a patient, with a darker line marking the median.

Implanted abnormalities were typically located in the ipsilateral hippocampus (30%) and the anterior middle temporal gyrus (15%, Figure S5A). Regions identified as MRI abnormal and iEEG were most frequently the ipsilateral hippocampus (7%, Figure S5C) and the ipsilateral fusiform gyrus (5%). Both implantation and abnormality difference in concordance was statistically tested across seizure outcome groups in the main text.

Patients with ILAE${}_{1,2}$ outcomes showed greater overlap between iEEG implantation and MRI abnormalities than those with ILAE${}_{3+}$, as confirmed by statistical testing (see Figure S5B, main text and Figure 4). A similar pattern was observed for the overlap between MRI and iEEG abnormalities (see Figure S5D, main text and Figure 5). These results suggest no outcome group bias and support the potential of the MRI only method to guide implantation.

# Leave-one-out cross-validation analysis on MRI-iEEG outcome differentiation


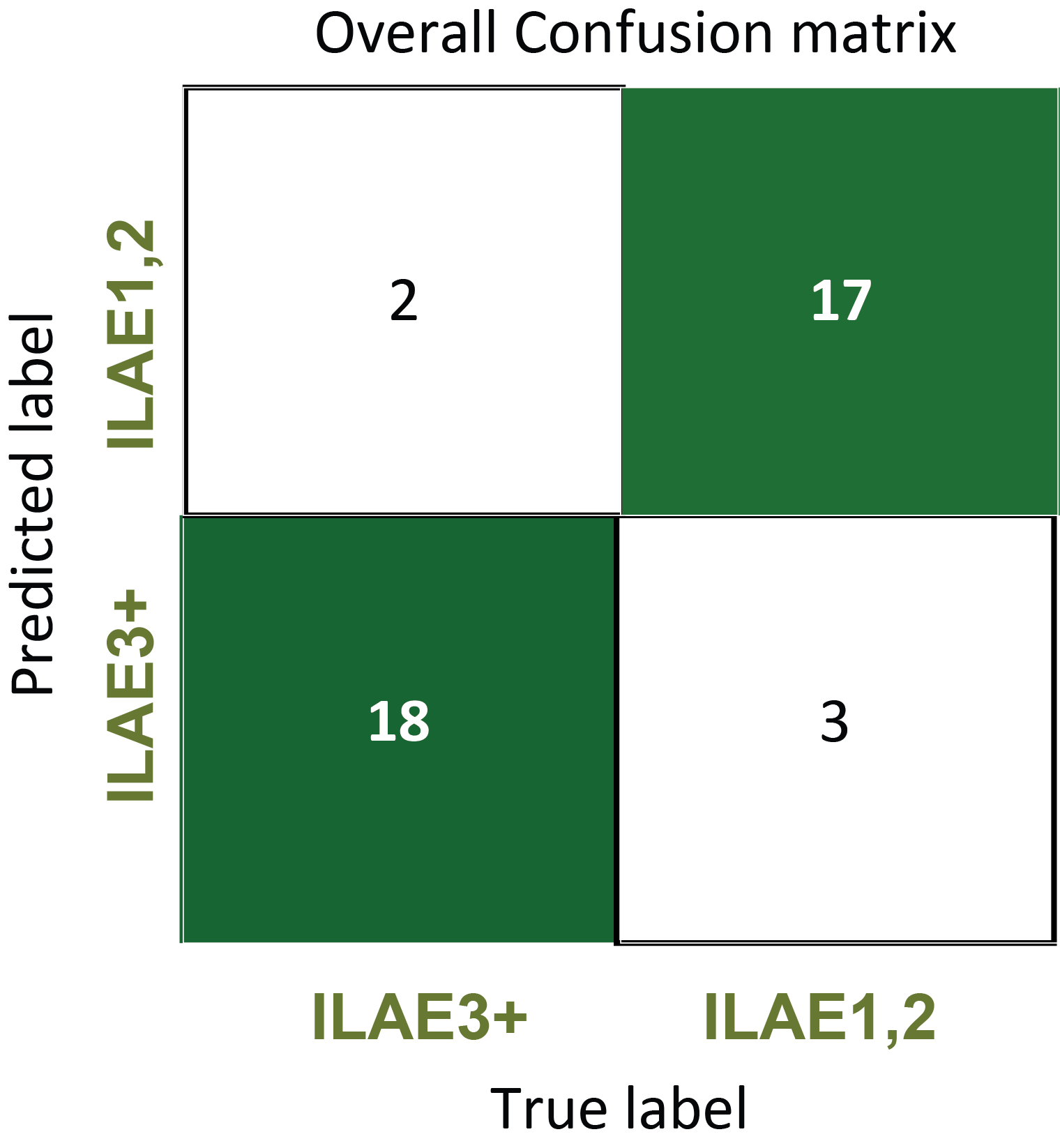


**Figure S6: Out-of-sample performance using leave-one-out cross-validation (LOOCV).** Confusion matrix summarizing the final out-of-sample predictions, including classification accuracy, sensitivity, and specificity.

To assess out-of-sample performance, we performed leave-one-out cross-validation (LOOCV). The overall confusion matrix (Figure S6) summarizes sensitivity (SEN = 0.84), specificity (SPE = 0.86), and accuracy (Balanced ACC = 0.85). This validation addresses potential overfitting while preserving the study’s original aim of differentiating seizure outcome groups, not building a predictive model.

# Bilateral implantation

We examined bilateral electrode coverage across all patients. Patients were considered to have bilateral iEEG if electrodes were present in both left and right hemispheres. We did not have post-surgical information to identify cases where independent bilateral temporal onsets among surgical failures (ILAE 3+). Table S7.1 summarizes the number of patients with bilateral iEEG by outcome group. We found that there is no significant difference between outcomes regarding the presence of bilateral implantations (X^2^ = 1.16, p = 0.29).

**Table S7.1:** Bilateral implantation per outcome at year 1

|  | $ILAE_{1,2}$ | $ILAE_{3+}$ | Test statistic |
| --- | --- | --- | --- |
| Bilateral implantation | 4/19 | 1/21 | X^2^ = 1.16, p = 0.29 |

# Localization of residual seizure focus

Patient level data showing the putative location of residual seizure focus for all patients’ outcomes at one year. Regions are classified as ipsilateral peri-resection cortex, contralateral corresponding cortex, or indeterminate. We found that there is no significant difference between outcomes in the ipsilateral peri-resection cortex (W = 149, p = 0.165) or the contralateral corresponding cortex (W = 196, p = 0.926), while there is in the indeterminate regions (W = 331, p < 0.01) (see Figure S8).

**Table S8.1:** Percentage of the putative location of residual seizure focus

| ID | Ipsilateral  peri-resection cortex | Contralateral  corresponding cortex | Indeterminate | ILAE Year 1 |
| --- | --- | --- | --- | --- |
| 1 | 3.23% | 0.00% | 6.50% | ILAE${}_{3+}$ |
| 2 | 19.35% | 0.00% | 3.00% | ILAE${}_{3+}$ |
| 3 | 0.00% | 9.68% | 3.25% | ILAE${}_{3+}$ |
| 4 | 3.23% | 6.45% | 3.00% | ILAE${}_{3+}$ |
| 5 | 9.68% | 3.23% | 3.50% | ILAE${}_{3+}$ |
| 6 | 19.35% | 3.23% | 1.75% | ILAE${}_{3+}$ |
| 7 | 6.45% | 0.00% | 2.00% | ILAE${}_{3+}$ |
| 8 | 0.00% | 0.00% | 4.00% | ILAE${}_{3+}$ |
| 9 | 6.45% | 3.23% | 3.50% | ILAE${}_{3+}$ |
| 10 | 3.23% | 3.23% | 2.75% | ILAE${}_{3+}$ |
| 11 | 3.23% | 0.00% | 3.50% | ILAE${}_{3+}$ |
| 12 | 3.23% | 0.00% | 2.75% | ILAE${}_{3+}$ |
| 13 | 0.00% | 3.23% | 4.50% | ILAE${}_{3+}$ |
| 14 | 3.23% | 3.23% | 3.25% | ILAE${}_{3+}$ |
| 15 | 3.23% | 0.00% | 6.00% | ILAE${}_{3+}$ |
| 16 | 0.00% | 3.23% | 2.75% | ILAE${}_{3+}$ |
| 17 | 6.45% | 3.23% | 4.00% | ILAE${}_{3+}$ |
| 18 | 0.00% | 3.23% | 3.25% | ILAE${}_{3+}$ |
| 19 | 3.23% | 3.23% | 4.00% | ILAE${}_{3+}$ |
| 20 | 0.00% | 3.23% | 3.25% | ILAE${}_{3+}$ |
| 21 | 6.45% | 6.45% | 2.25% | ILAE${}_{3+}$ |
| 22 | 9.68% | 0.00% | 2.75% | ILAE${}_{1,2}$ |
| 23 | 35.48% | 0.00% | 1.50% | ILAE${}_{1,2}$ |
| 24 | 0.00% | 9.68% | 2.00% | ILAE${}_{1,2}$ |
| 25 | 0.00% | 0.00% | 2.75% | ILAE${}_{1,2}$ |
| 26 | 16.13% | 0.00% | 1.00% | ILAE${}_{1,2}$ |
| 27 | 9.68% | 6.45% | 1.00% | ILAE${}_{1,2}$ |
| 28 | 12.90% | 0.00% | 2.75% | ILAE${}_{1,2}$ |
| 29 | 0.00% | 6.45% | 2.50% | ILAE${}_{1,2}$ |
| 30 | 6.45% | 6.45% | 2.75% | ILAE${}_{1,2}$ |
| 31 | 0.00% | 6.45% | 2.50% | ILAE${}_{1,2}$ |
| 32 | 19.35% | 6.45% | 2.75% | ILAE${}_{1,2}$ |
| 33 | 3.23% | 3.23% | 2.75% | ILAE${}_{1,2}$ |
| 34 | 3.23% | 0.00% | 2.75% | ILAE${}_{1,2}$ |
| 35 | 19.35% | 0.00% | 3.75% | ILAE${}_{1,2}$ |
| 36 | 3.23% | 0.00% | 1.75% | ILAE${}_{1,2}$ |
| 37 | 6.45% | 3.23% | 1.75% | ILAE${}_{1,2}$ |
| 38 | 9.68% | 6.45% | 1.75% | ILAE${}_{1,2}$ |
| 39 | 9.68% | 3.23% | 3.25% | ILAE${}_{1,2}$ |
| 40 | 0.00% | 0.00% | 2.25% | ILAE${}_{1,2}$ |


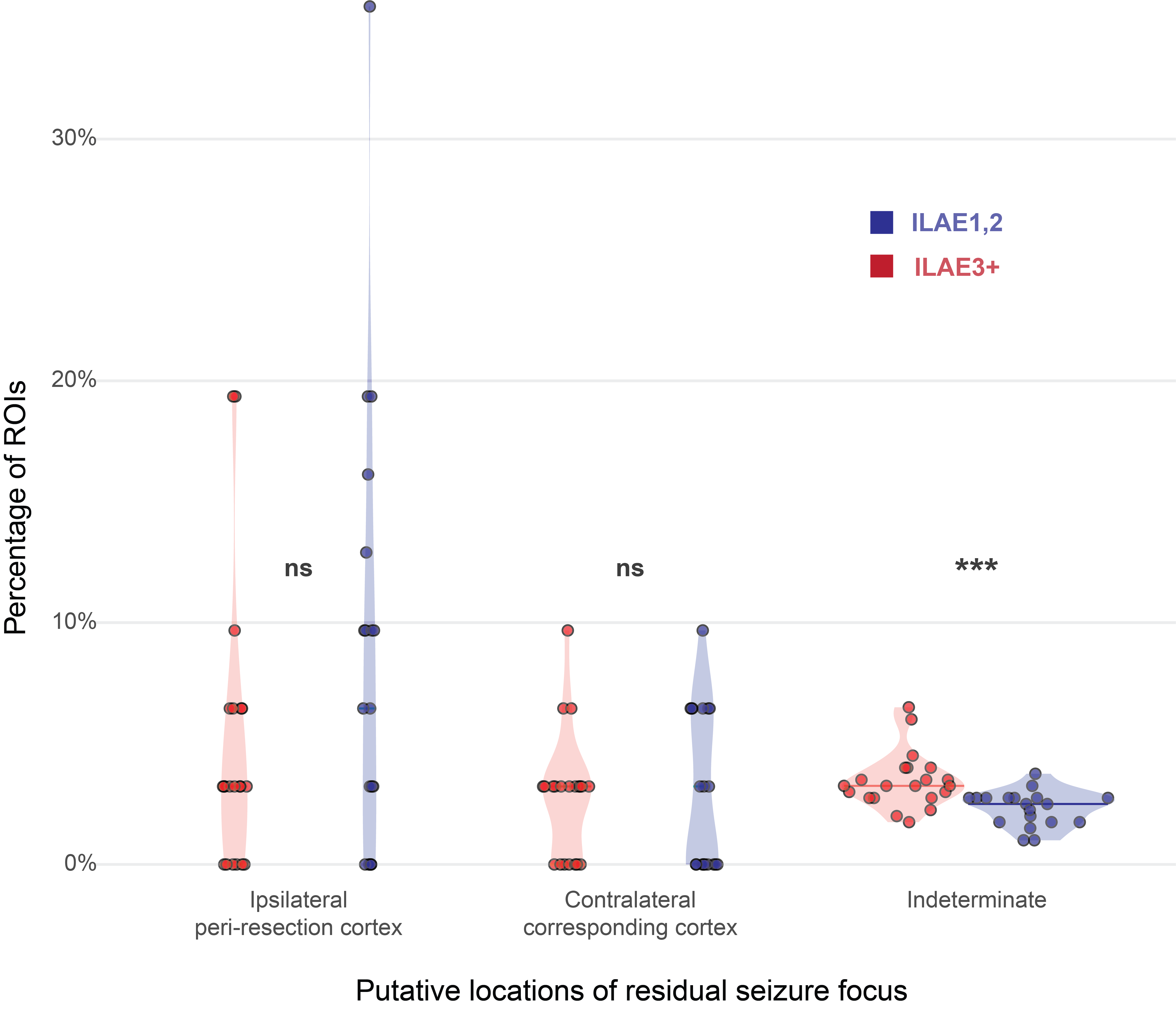


**Figure S8: Percentage of putative residual seizure focus locations:** Categorized as ipsilateral peri-resection cortex, contralateral corresponding cortex, or indeterminate, and separated by outcome. Each point represents a patient, with a darker line marking the median.

# Overlap of iEEG abnormal and MRI normal regions


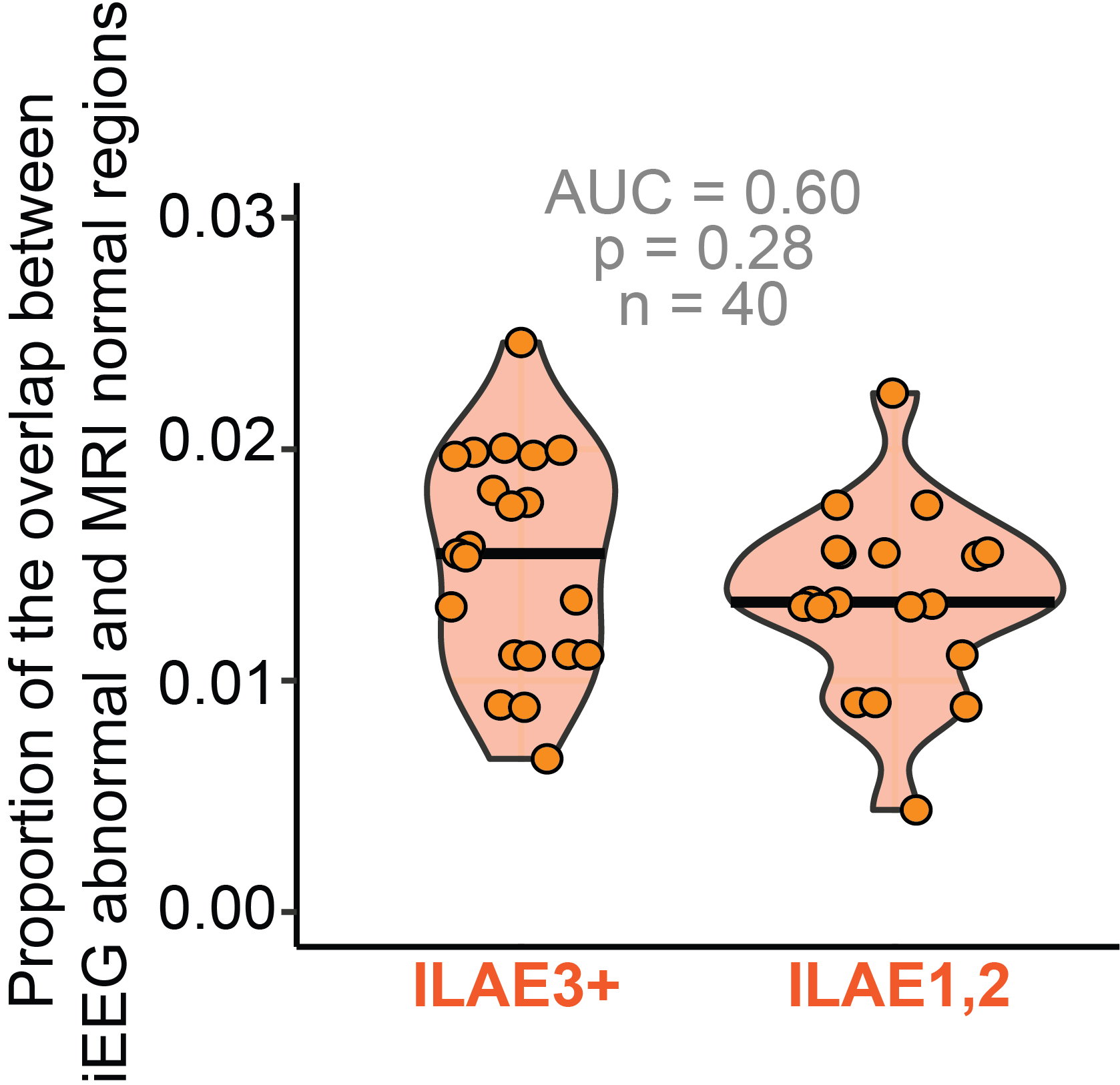


**Figure S9: Proportion of the overlap between iEEG abnormal and MRI normal regions**: Distribution is separated by outcome. Each point represents a patient, with a darker line marking the median.

We found that only a small proportion of iEEG abnormalities were in MRI-normal areas, with a relatively larger number observed in patients with ILAE 3+ outcomes, and this measurement does not separate outcome reliably (AUC = 0.60, p = 0.28) (see Figure S9).

Distribution of z-scores in SWM and GM


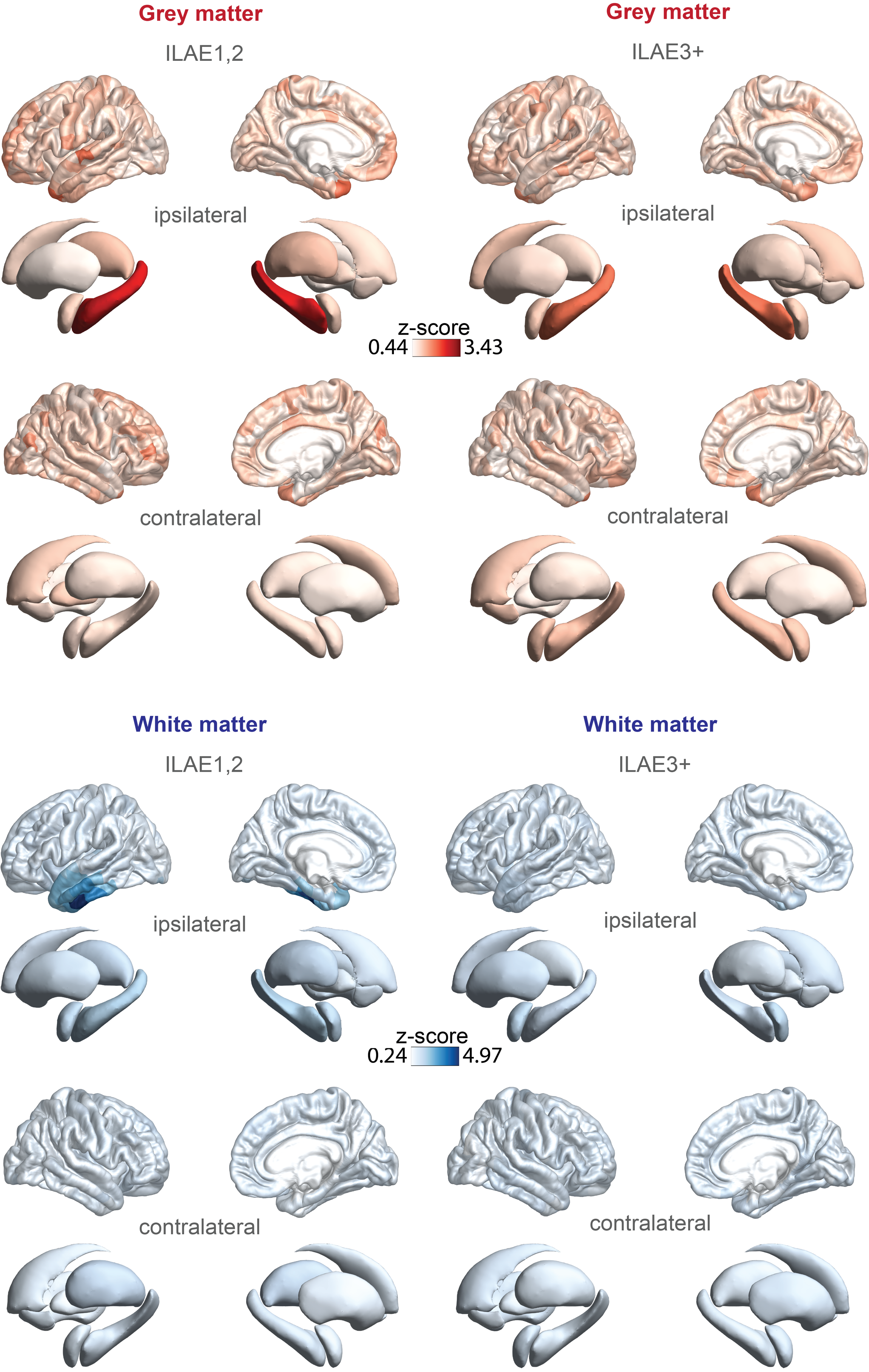


**Figure S10: Spatial distribution of absolute z-scores across the cohort in SWM and GM broken down by outcome**.

We found that absolute z-scores were more widely distributed and, on average, lower in patients with ILAE 3+ outcomes compared to those with ILAE 1,2. In ILAE 1,2 patients, the highest z-scores were concentrated in the ipsilateral anterior temporal lobe (in SWM) and the hippocampus (in GM), identifying these regions as key targets for resection.
